# Successful gel-immersion cannulation of hepaticojejunostomy during enteroscopy-assisted endoscopic retrograde cholangiopancreatography

**DOI:** 10.1055/a-2980-7307

**Published:** 2026-10-27

**Authors:** Shintaro Kawasaki, Atsuto Kayashima, Haruka Okada, Hiroki Tamagawa, Masayasu Horibe, Takanori Kanai, Motohiko Kato

**Affiliations:** 1 Center for Diagnostic and Therapeutic EndoscopyKeio University School of MedicineTokyoJapan; 2 Division of Gastroenterology and Hepatology, Department of Internal MedicineKeio University School of MedicineTokyoJapan

Hepaticojejunostomy stricture after Whipple surgery is one of the common late postoperative complications. 1​ Enteroscopy-assisted– endoscopic retrograde cholangiopancreatography (EA-ERCP) has become a first-line minimally invasive approach for the management of benign biliary obstruction in patients with surgically altered anatomy. 2​ ​3​ However, postoperative scarring and anatomical distortion around the hepaticojejunostomy often make the identification of the anastomotic site and selective biliary cannulation technically challenging. Because the short-type single-balloon enteroscope (SIF-H290s, Olympus, Tokyo, Japan) was not equipped with a water jet function, we introduced the viscous gel immersion technique by delivering Viscoclear (Otsuka Pharmaceutical Factory, Tokushima, Japan) through a BioShield irrigator (US Endoscopy, Mentor, OH, USA) 4​ ​5​ during EA-ERCP.

A 60-year-old man developed a benign anastomosis stricture and cholangitis after Whipple surgery for a duodenal neuroendocrine tumor. An enteroscope was advanced to the suspected hepaticojejunostomy site. However, maintaining a stable endoscopic view of the anastomosis was difficult because of postoperative scarring and anatomical distortion ( Fig. 1a , Video 1 ). The gel immersion technique during EA-ERCP offered clear visualization of the pinhole-like hepaticojejunostomy ( Fig. 1b ). In addition, contact between the endoscope or the guidewire and the anastomosis caused intermittent bleeding; however, repeated injection of the gel effectively washed away the blood and maintained a clear endoscopic field throughout the procedure. A guidewire was successfully advanced into the bile duct ( Fig. 2 a, b ), after which the anastomotic stricture was incised using a precut knife over the guidewire. Balloon dilation was subsequently performed ( Fig. 2c ), followed by the successful placement of a 5-Fr endoscopic biliary stent ( Fig. 2d ). The patient experienced no procedure-related adverse events and showed complete resolution of cholangitis. This gel immersion method offered two benefits. Firstly, luminal decompression enabled stable scope movement. Secondly, gel-mediated hemostasis and flushing improved visualization of bleeding. Gel immersion cannulation during EA-ERCP may facilitate the successful cannulation of highly challenging hepaticojejunostomies.

**Fig. 1:**
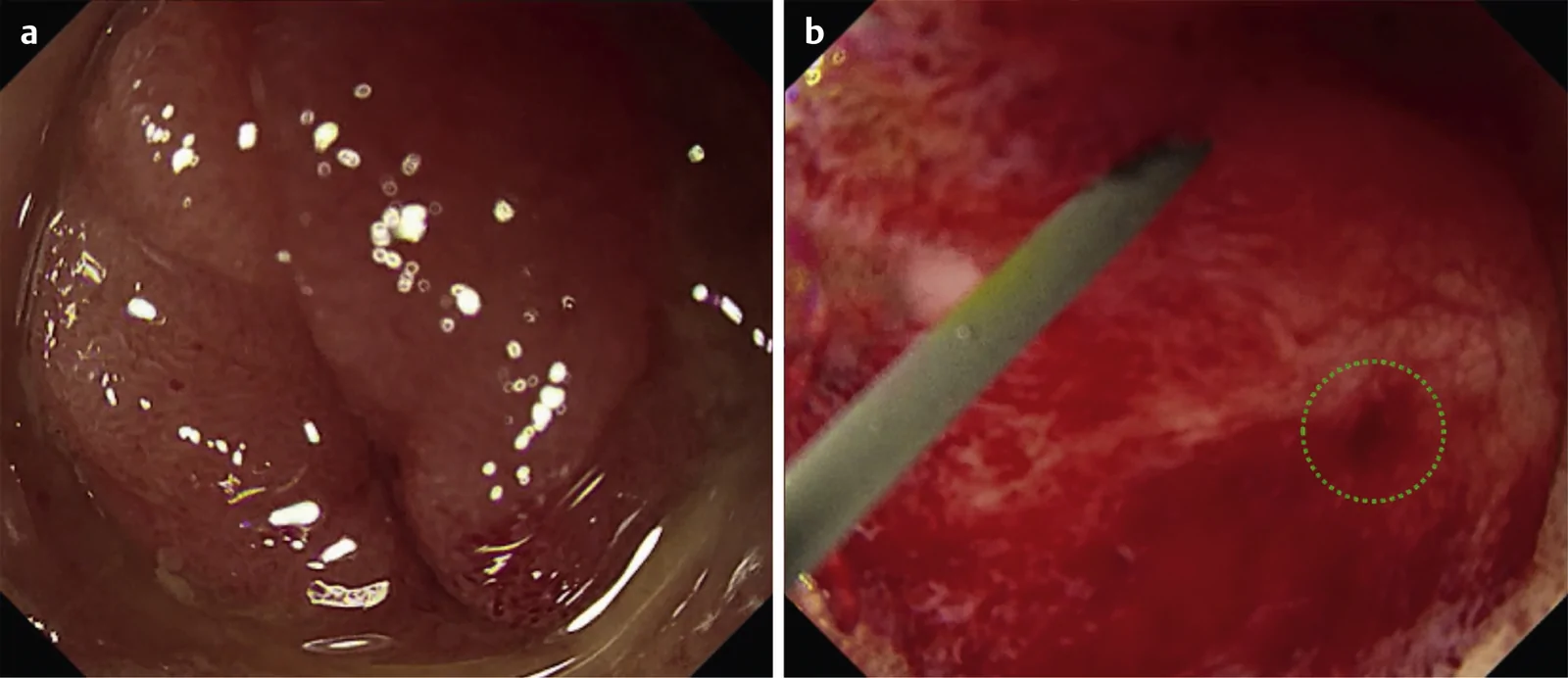
( **a** ) Under CO _2_ insufflation, the hepaticojejunostomy was poorly visualized. ( **b** ) The gel immersion technique during EA-ERCP provided a stable endoscopic view of the hepaticojejunostomy. The green circle indicates the hepaticojejunostomy.

**Video 1** Successful gel-immersion cannulation of hepaticojejunostomy during balloon-assisted enteroscopy. The video link below:


**Fig. 2:**
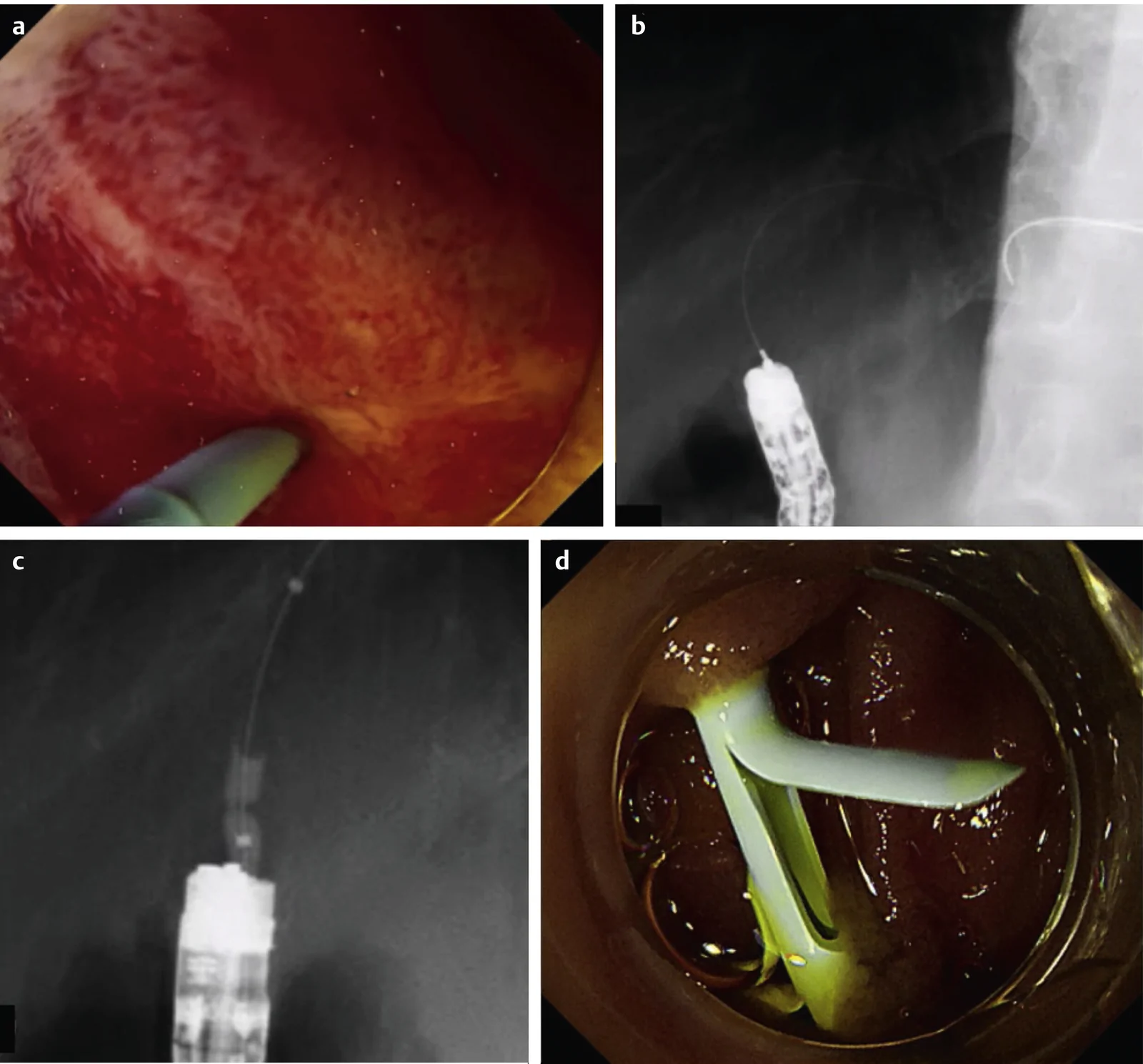
( **a** and **b** ) Successful guidewire insertion through the pinhole-like hepaticojejunostomy. ( **c** ) Additional balloon dilation. ( **d** ) 5Fr endoscopic biliary stenting.

Endoscopy_UCTN_Code_TTT_1AR_2AG

## Declaration of the of GenAI Use

The authors declare that no generative artificial intelligence was used in the preparation of this manuscript.
